# NetView: A High-Definition Network-Visualization Approach to Detect Fine-Scale Population Structures from Genome-Wide Patterns of Variation

**DOI:** 10.1371/journal.pone.0048375

**Published:** 2012-10-31

**Authors:** Markus Neuditschko, Mehar S. Khatkar, Herman W. Raadsma

**Affiliations:** Reprogen – Animal Bioscience, Faculty of Veterinary Science, University of Sydney, Camden, New South Wales, Australia; University of Bristol, United Kingdom

## Abstract

High-throughput sequencing and single nucleotide polymorphism (SNP) genotyping can be used to infer complex population structures. Fine-scale population structure analysis tracing individual ancestry remains one of the major challenges. Based on network theory and recent advances in SNP chip technology, we investigated an unsupervised network clustering method called Super Paramagnetic Clustering (Spc). When applied to whole-genome marker data it identifies the natural divisions of groups of individuals into population clusters without use of prior ancestry information. Furthermore, we optimised an analysis pipeline called NetView, a high-definition network visualization, starting with computation of genetic distance, followed clustering using Spc and finally visualization of clusters with Cytoscape. We compared NetView against commonly used methodologies including Principal Component Analyses (PCA) and a model-based algorithm, Admixture, on whole-genome-wide SNP data derived from three previously described data sets: simulated (2.5 million SNPs, 5 populations), human (1.4 million SNPs, 11 populations) and cattle (32,653 SNPs, 19 populations). We demonstrate that individuals can be effectively allocated to their correct population whilst simultaneously revealing fine-scale structure within the populations. Analyzing the human HapMap populations, we identified unexpected genetic relatedness among individuals, and population stratification within the Indian, African and Mexican samples. In the cattle data set, we correctly assigned all individuals to their respective breeds and detected fine-scale population sub-structures reflecting different sample origins and phenotypes. The NetView pipeline is computationally extremely efficient and can be easily applied on large-scale genome-wide data sets to assign individuals to particular populations and to reproduce fine-scale population structures without prior knowledge of individual ancestry. NetView can be used on any data from which a genetic relationship/distance between individuals can be calculated.

## Introduction

Genetic variation among and within populations arises from many factors such as mutation, migration, bottlenecks and admixture, and carries the signatures of random drift and natural selection. The complex interplay of these forces may give rise to the formation of populations which are genetically sub-structured. Uncovering such population sub-structures can give insight into the history and evolution of populations. Furthermore, identifying population sub-structures and assigning individuals to sub-populations is potentially useful in association studies, where sub-structures have to be accounted for to reduce spurious associations [1] and provide useful information for conservation of genetic resources [2]. Rapid innovations in high-throughput sequencing and array technologies have led to the identification of large numbers of SNPs and to the development of SNP chips in a number of species [3], [4], [5]. These developments are providing new biological insights into complex population structures [6]. There is also growing interest to detect population sub-structures in “unrelated” samples in humans [7], [8], [9], livestock [10], [11], [12], dogs [13] and other biological populations (e.g. plants [14]). An efficient analytical tool for population structure analysis would utilise all available high-density SNP data to identify sub-structures in the absence of prior ancestry information.

To date, the two most common approaches for identifying population structure and assigning individuals to their population of origin are based on methods derived from PCA [15] and model-based algorithms implemented in programs such as Structure [16], [17], Admixture [18] and more recently fineStructure [19]. As discussed below, these two approaches have limitations in their prior assumptions, their capacity to handle large-scale genomic data or in visualizing and interpreting analysis outputs.

PCA is a classical non-parametric linear reduction technique for uncovering population structure by arranging all principal components (PCs) according to the explained variance. Based upon this principle, two different PCA approaches have mainly been used. In population-based association studies, PCA uses covariance among normalized genotype scores of samples to correct for population stratification [20], [21], whilst in population genetics, Principal Coordinate Analysis (PCoA) as well as Multidimensional Scaling (MDS) are applied, which utilize allele sharing distances (ASD) between samples to infer population clusters [12], [22], [23]. As such, PCA is not capable of assigning samples to clusters nor estimating number of clusters (K), hence this analysis is commonly applied in combination with standard clustering tools, e.g. *k*-means [24] and Model-based Clustering (Mclust) [25] to allocate the individuals to their “true” cluster [26]. In order to determine the optimal number of clusters with *k*-means and Mclust different criterions are applied e.g. Calinski criterion [27] and Bayesian Information Criterion (BIC) [28]. It has been shown that this two-stage strategy is a powerful tool for the identification of population clusters using genome-wide SNP data sets including thousands of individuals and many thousands of SNPs [29]. However, this approach is less useful for visualization of population data sets which consist of closely related sub-populations [30]. In such population data sets, the commonly applied PCA-scatter plots (i.e. top 1–3 PCs) can highlight the difference of the most distinct sub-populations but could be limiting in separating the closely related sub-populations [31]. The number of PCs can be increased [32] and genetically distinct sub-populations can be excluded in an iterative process to increase the resolution of the PCA-scatter plot [30], [33]. Since visualization at higher than 3-D space is not effective, the interpretation of PCA-based methods becomes difficult and limited.

In contrast to PCA-based methods the program Structure uses a model-based algorithm to identify population structure. This method has been used in numerous population structure studies [34], [35]. However, the detailed modelling of the data causes intensive computational demands [21], [26], [36], making this algorithm impractical to apply on high-density SNP data sets. Recently, alternative model-based algorithms as implemented in the programs Admixture [18] and *frappe* [37] have been used for uncovering genome-wide population structures [38], [39]. These algorithms are computationally efficient and can be easily applied on genome-wide SNP data sets to infer individual ancestry. However, these methods are still sensitive to the nature of sample collection (e.g. inclusion of closely related individuals) and therefore require a data-validation check. Commonly this data-validation check is done by calculating identity-by-descent (IBD) for all pairs of individuals and removing one individual from each pair with a proportion IBD above some threshold (e.g. IBD more than 0.125 [40]). Furthermore, current methods (PCA- and model-based approaches) infer population structures, supposing that no sub-structures exist within the populations. As sub-structures frequently exist within populations, these methods do not represent any information on the relatedness of individuals within the population, for example in the analyses of populations with strong founder effects and closely related individuals.

Recently, we and others have shown that network theory can be used for uncovering fine-scale population structure [41], [42]. Network-based procedures sub-divide the population into a network of nodes or community structures, based on the density of the connections within and between different sub-groups, which provides a means to identify and visualize the structure of the respective global population. Network analyses have been successfully applied in a wide variety of contexts, e.g. in the analysis of epidemiology [43], in identifying graph structures in the Internet and world wide web [44] and for deriving community structures in social and biological networks [45]. To identify these community structures, many different algorithms have been developed, including vertex similarity [46], the vertex degree gradient [47], the resistor network [48] and the Potts Hamiltonian Model [49].

Here, we describe the application of an unsupervised network clustering-method, so called Super Paramagnetic Clustering (Spc) as implemented in the software program Sorting Points into Neighbourhood (Spin) [50], [51], which uses the Potts Hamiltonian Model to identify stable clusters in networks [52]. We evaluate the performance of Spc extensively on three previously described data sets: simulated [19], human [53] and cattle [31]. We demonstrate the utility of Spc to detect complex population structures using very dense SNP genotype data, to assign individuals to sub-populations and to determine the optimum number of clusters. To our knowledge this is the first application of Spc to large-scale genomic data for population applications.

We integrated Spc output into high resolution visualisation programmes and constructed a population analysis pipeline which we call “NetView”. We show that it is feasible to visualize and detect fine-scale population structure as well as the genetic relatedness of individuals, thereby detecting important key individuals (highly connected individuals) and less significant individuals (less connected individuals) from “unrelated” samples of a population.

## Methods

### Development of NetView

NetView, a high-definition network visualization, is an analysis pipeline which consists of 5 distinct components: **(A)** data preparation and editing, **(B)** calculation of a genetic-relationship or genetic-distance matrix among all individuals/samples, **(C)** network construction, **(D)** clustering of individuals within the population network, and finally **(E)** network-based visualization of the clustering results.

The NetView procedure is shown schematically in Figure S1 and at http://sydney.edu.au/vetscience/reprogen/netview.

#### A: Data preparation and editing

NetView can handle any data from which a relationship or distance between pairs of individuals can be estimated. Genotypic data from SNP chips now presents the typical data input of choice. Such data sets often require editing and quality control (QC) filtering in order to minimize spurious artefacts associated with genotyping. Many proprietary scripts or public domain software packages are available for this purpose. We frequently use Plink as described in its manual (http://pngu.mgh.harvard.edu/~purcell/plink/) [54]. Typically we check data for the presence of duplicate samples by comparing genotype similarity among all samples and remove one of each duplicate. In general, SNP arrays do not call all SNPs with equal efficiency or completeness, and we remove SNPs and samples with a call rate <90%. The SNPs with extreme deviation from Hardy Weinberg Equilibrium (HWE), assessed using P-values <0.001 in Fisher’s exact test, and with very low minor allelic frequency <0.01 (MAF) are excluded from the analysis to provide “common” well-defined, non-duplicated SNPs for the calculation of the relationship matrix. Our preference is to use SNPs only with known positions although this is not essential; it is a prerequisite only if haplotype-based similarity is the basis for estimating relationships. The exact thresholds for different inclusion criteria can be modified for different data sets, as described below.

#### B: Computation of genetic distance matrices

NetView requires an input matrix of genetic relationships or distances between all individuals in the network construction step. The most frequently used methods to determine genetic distances among individuals in diversity studies are genetic model-based distances (e.g. Wright’s F_ST_ [55] and Nei’s standard genetic distance [56]) as well as ASD [57]. Model-based distances quantify the extent of genetic differentiation among individuals based upon the allele frequencies of marker genotypes within predefined sub-populations. Hence, in all studies in which model-based distances are applied, knowledge of individual ancestry is a prerequisite. Contrary to model-based distances, computation of classical ASD is only based on genotypic or haplotype information [54]. Commonly, genome-wide ASD are calculated as one minus the average proportion of shared alleles, which can be identical by state (IBS) or descent (IBD) as obtained through Plink [54] or similar [19]. It is common for population structure analyses to rely on ASD derived from IBS-based relationships [12], [53]. We exemplify NetView based on ASD derived from IBS [54] and shared haplotypes [19]. However, as explained above, NetView can also be applied on model-based distances.

#### C: Network construction based on genetic distances

Given the pair-wise distances between all individuals (*D*), a fully connected population network is created using Spc [50]. In this network individuals are considered as nodes, and connections between a pair of individuals as edges. In population networks the thickness of edges are used to reflect the genetic distance between individuals as described below. Spc computes such a network using an algorithm which requires the specification of the maximum number of nearest neighbours (*k*-NN) an individual can have. Based on the *k*-NN specification, the pair-wise distances between nearest neighbours and the distances of these neighbours to all other individuals in the data set are considered. Individuals without a nearest neighbour in the population network are then connected to individuals, which have the smallest genetic distance with the individual in question. The topological structure of the population network generated by this procedure depends on the parameter *k*-NN. There is no suitable objective procedure to optimize *k*-NN. We set k-NN = 10 as the default value, as suggested by previous applications [50], and also recommend exploring lower *k*-NN for very small sample collections (i.e. data sets with less than 10 samples per sub-population). The network construction is described in detail in Blatt *et al.* [50] and Text S1.

#### D: Clustering of individuals within the population network

In order to interpret the existence of sub-populations within the population network, all individuals get clustered based on genetic distance by an unsupervised clustering procedure. Here also Spc is used in a model based simulation framework (described in Blatt *et al.* [50] and Text S1) which generates clusters at different thresholds of genetic distance (Note that here genetic distance is analogous to the temperature gradient used in the detailed description of the method and as applied to physical analysis or material stability). The optimum threshold and corresponding equilibrium state, so called “super-paramagnetic state”, is identified by the algorithm itself by measuring the variation in cluster size along a gradient of thresholds of genetic distance [58]. The state where the size and hence number of clusters is stabilized is determined by a Hamiltonian cost function as described in detail in Text S1 and Blatt *et al.* [50]. The results of the analyses at different thresholds of genetic relationship are combined and provide the hierarchical clustering of all individuals. We term the thresholds of genetic distance at which a cluster configuration (size and number) is identified as the cluster stability (*CS*). Consequently the resultant hierarchical population structure can be described as the branching of identified populations at a specific *CS*. Branching at high *CS* signifies clusters with strong internal genetic relationships, whereas clusters defined with low *CS* indicate less stable population structures which may arise from either internal sub-structures or a high number of external links to other populations.

The main output from Spc consists of a re-ordered distance matrix of all individuals based on the final optimal clustering, and a hierarchical tree of cluster relationships. Spc provides the clustering relationships in the form of a binary matrix for presence or absence of a connection between any pair of individuals in the data set. This binary relationship matrix is multiplied with the relationship matrix, to define the strength between connected individuals i.e. thickness of the edges. This cluster relationship matrix is then visualized in the network visualization step.

#### E: Network visualization following clustering

In NetView we have applied open source software Cytoscape (http://www.cytoscape.org) [59] for visualizing the final network which is based on the cluster relationship matrix from Spc as described in the previous section. We used the network analysis tools software (NeAT) [60] to transform the cluster relationship matrix into a format required by Cytoscape where, population structure is now represented in terms of nodes, edges between nodes and thickness of edges. In the final network presentation, the relationships between the individuals within a cluster are described such that more closely related individuals are co-located, and distantly related individuals are separated. In addition the thickness of an edge expresses the strength of relationship in proportion to the genetic distance between two nodes. Similarly, in general more closely related clusters are co-located on the network as a whole but the separation is not to scale in relation to the genetic distance (For the actual hierarchal relationship between clusters the Spc tree is more informative). Overall the network visualized shows detailed relationships among individuals grouped within and between populations and provides a high-definition network visualization (NetView) of the populations. Important key individuals within populations can easily be detected by the node size which is based on the number of direct connections to the node (degree centrality). It should be noted, that using Cytoscape different visualization styles for networks can be applied. For population networks we found it more attractive to apply the circular and organic layout style. Using the circular visualization style the relationship between individuals and their connectivity can be easily investigated, whilst the organic visualization provides a more meaningful NetView for closely related populations and populations containing sub-structures.

## Results

### Application of NetView

We demonstrate the application and utility of NetView in three population data sets. The first data set is a simulated population of 400 individuals genotyped for 2.5 million SNPs where the relationship between individuals is known and predetermined [19]; the second is a typical human data set related to diversity and HapMap studies where the data set comprises a relatively small number of populations, a large effective population size (N_e_), limited natural selection, and high-density SNP genotypes [53]. For the third and final data set we have chosen a cattle population diversity HapMap data set where many breed groups exist, most with a relatively small N_e_, intense directional selection, and genotyped for relatively low-density genome-wide SNP profiles [31]. In order to provide meaningful comparisons of the output generated under NetView in each application, we used equivalent relationship input matrices applied in the previously published studies (i.e. shared haplotypes for the simulated data set [19], and genotype-based IBS for the human and bovine sample collection [31], [53]).

#### A: Simulated population data set

**Figure 1:**
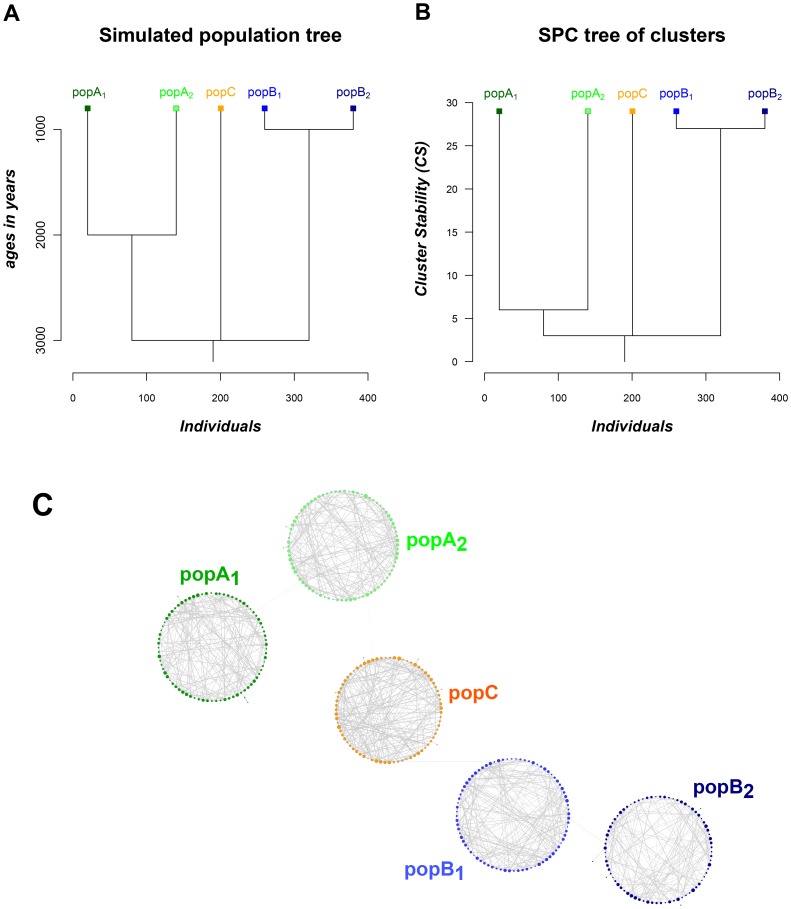
Spc and NetView analysis of the simulated data set. (**A**) Population structure used for creating the simulated data (adapted from Lawson *et al.* [19]). (**B**) Spc tree of clusters representing the grouping of individuals with *k*-NN = 10. The individuals have been separated into 5 clusters, representing the three main populations and the additional existence of two sub-populations (PopA_1_ and PopA_2_, PopB_1_ and PopB_2_). Each cluster is represented by a box; with Y axis positions indicating the stability of each cluster, whilst the X-axis positions are indicating the proximity between clusters. (**C**) High-definition network visualization (NetView) of the simulated population structure. Each individual is represented by a node; with the different shades denote the sample origin. The thickness of edges varies in proportion to the genetic distance and has been used to visualize individual relationships within and between populations. The node size varies in proportion to the numbers of edges per node, and illustrates how well each individual is connected within the population.

**Figure 2:**
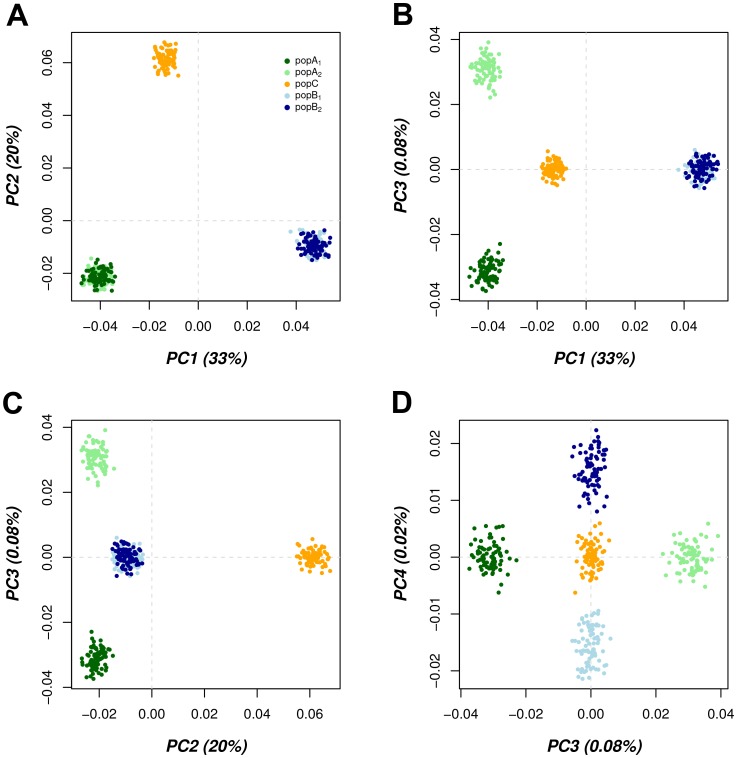
PCA scatter plots of the simulated data set. Projection of individuals from 5 populations onto a two dimensional (X,Y) subspace of four PCs. The panels **A** to **D** show pair wise comparison of PC combinations. Each individual is represented by a datum point. Each sub-population is denoted by a separate colour. The variation captured by each PC is indicated in parenthesis next to the axis label.

**Figure 3:**
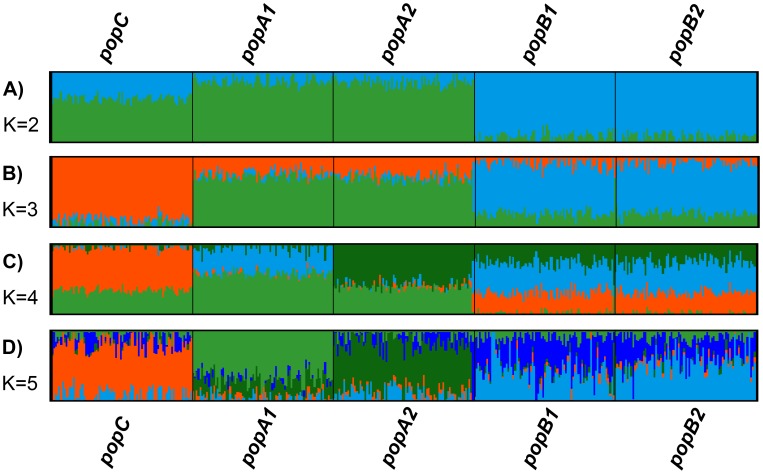
Cluster assignment of the simulated population data following analysis by Admixture using 2–5 clusters (K). Individuals are presented by a single vertical column divided into K colours. Each colour represents one cluster, and the length of the coloured segment corresponds to the individuals estimated proportion of membership in that cluster. For each K, 10 iterations were performed. The panels **A** to **D** represent the cluster patterns at K = 2 to 5.

The simulated data used in this study reflects real human population structures as presented and analysed by Lawson *et al.* [19]. To imitate these population structures, the simulation starts with a high N_e_ (N_e_ = 5000) and an exponential growth rate until present day. To simulate the present N_e_ ∼ 3100 [61], N_e_ drops through a bottleneck. After this demographic event the population splits into three populations (PopA, PopB, PopC) 3000 years ago, whilst two populations additionally split 2000 (PopA_1_ and PopA_2_) and 1000 (PopB_1_ and PopB_2_) years ago respectively (Figure 1A). It was assumed that one generation was 26 years. From the final five populations, data on 80 samples from each population were used in this study. The same recombination maps as described in Phase II Hapmap [62] were used to simulate 100 regions, each spanning 5 Mb. There were about 2.5 million SNPs after considering SNP alleles observed in more than one individual. We used the haplotype-based relationship matrix between individuals from these data as provided by Lawson *et al.* [19], where patterns of haplotype similarity of each individual are used to reconstruct the pair-wise relationship between all individuals along each chromosome. The final genome-wide “co-ancestry matrix” is the average of the results along each chromosome, and provides the input matrix on the ancestral relationship between all individuals in the population. It should be noted that PCA and Admixture results of the simulated data set as presented in Figures 2 and 3 were also performed and published by Lawson *et al.* [19] on a slightly different sample collection. Recently Lawson *et al.* [19] analysed this data with the software fineStructure [19] that provides a tree-like representation of the data which can be compared to Spc’s output tree.

#### NetView

Applying Spc on the simulated data every individual has been allocated to its correct sub-population. A hierarchical tree of clusters obtained from the analysis of the entire simulated data set is presented in Figure 1B. As shown in the figure, the Spc tree starts to split into three major groups and ends up with a final organization of clusters, where all individuals have been separated to each of the five single sub-populations along a continuous gradient of cluster stability. The shape of the hierarchical tree observed reflects the simulated population scenario, by separating the populations into the three main populations including a further differentiation of PopA and PopB into the respective sub-populations at more recent time events. The hierarchical structure of the tree shows that the sub-populations PopA_1_ and PopA_2_, and PopB_1_ and PopB_2_ are more closely related (Figure 1B). However, the tree reveals little information about the relationship between individuals within the sub-populations. In order to reveal this the high-definition network visualization of the simulated population structure was undertaken.

Under network visualization, each individual is presented by a node as shown in Figure 1C, with different shades denoting the origin of the samples. The thickness of edges, which varies in the proportion to the genetic distance, has been used to visualize individual relationships within populations. The node size, which varies in proportion to the numbers of edges per node (degree centrality) illustrates the connectivity of each individual within the population and shows a relatively greater or lesser genetic relatedness of the individual within the sub-population.

The topology of the network visualization highlights the existence of clusters of populations and additional expression of the fine-scale population structure within the identified sub-populations. Furthermore, the high-definition visualization shows that the majority of individuals of each population are highly interconnected, which indicates that no further sub-structures exist within the identified sub-populations. The values of degree centrality, quantifying the relative representation of each individual within the population, immediately highlights the connectivity of each individual, simultaneously revealing important key individuals and less connected “orphans”.

The results of NetView were compared with PCA results and Admixture on this data set as shown in Figure 2 and Figure 3.

#### PCA and *k*-means analysis

Given the genome-wide proportions of shared alleles between individuals (*A*), we directly applied PCA on *A* to compute its singular PCs. To determine the number of significant PCs we have used the empirical method Horn’s parallel analysis as implemented in the statistical programme package *paran* (http://www.r-project.org). This method employs Monte Carlo estimates to retain most significant PCs, according to the given significance level and number of iterations. Here, we chose a significance level of P = 0.01 and 10,000 iterations, which have been suggested in the modified version of *Horn’s* parallel analysis [63]. After determining the number of significant PCs, *k*-means clustering was applied on low-dimensional data (i.e. number of significant PCs) to allocate the individuals in the given number of clusters. In order to determine the optimal number of clusters we computed the Calinski criterion [27] as implemented in the statistical programme package *vegan* (http://www.r-project.org) for each K, increasing K from 2 to 7.

Horn’s parallel analysis resulted in 4 significant PCs accounting for 33%, 20%, 0.08% and 0.02% of the variation among all individuals. Individuals are projected onto the sub-space of the four significant PCs on a pair-wise comparison of the PCs where each individual is represented by a datum point, and each sub-population is denoted by a different colour. In contrasting PCA1 vs PCA2, the three founder populations PopA, PopB and PopC are clearly differentiated (Figure 2A) corresponding to the first tree branch in the unsupervised Spc analysis (Figure 1B). A clear differentiation between sub-population PopA_1_ and PopA_2_ occurred by contrasting PC1 vs PC3 (Figure 2B) and contrasting PC2 vs PC3 (Figure 2C) whilst the final contrast of PC3 vs PC4 showed the full separation of all five populations (Figure 2D), corresponding to the most recent branching of all populations in the Spc analysis (Figure 1B). The Calinski criterion clearly identified the true number of clusters (Figure S2). Using *k*-means clustering on the low-dimensional data (4 PCs) and the predetermined optimal number of clusters (K = 5) all individuals were correctly assigned to their respective sub-populations.

#### Admixture

We applied Admixture on the entire simulated data set for each value of K, increasing K = 2 to 5 (the true number of populations). Convergence between independent runs at the same K was monitored by comparing the resulting log-likelihood scores (LLS) following 10 iterations, and was inferred from stabilized LLS with less than 1 LL unit of variation between runs. Convergence was observed for all K values (K = 2 to 5). Ten-fold cross validation was performed and cross validation errors were computed to determine the optimal number of clusters as suggested by Admixture at each value of K.

Following cross-validation Admixture suggests an uppermost hierarchical structure of K = 1 and shows a clear point of change in the slope of the error rate by further increasing K to 3, however the error rate values from K = 1 to 5 were in a narrow range (Figure S3).

The assignment of individuals to separate clusters shows that Admixture successfully splits the three founder populations (PopA, PopB and PopC), but is unable to differentiate the more closely related sub-populations PopA_1_ and PopA_2_ and PopB_1_ and PopB_2_. For instance, given K = 2 and K = 3, Admixture confirms the findings of PCA on the first two significant PCs by allocating individuals from PopB to one and individuals from PopA and PopC to the other cluster (Figure 3A) and further separating PopC from PopA (Figure 3B). At K = 4 the sub-populations PopA_1_ and PopA_2_ become differentiated, with individuals from PopA_2_ associated with a high proportional membership of PopA_1_ (Figure 3C). Admixture could not differentiate populations PopB_1_ and PopB_2_ at any given K (Figure 3A-D).

To test the sensitivity of NetView to varying sample size, we further analysed different sub-sets of the data by reducing the number of individuals by randomly sampling 10, 20 and 40 individuals from each population. To imitate a heterogeneous sample collection, we additionally applied NetView on a data set consisting of 80 samples from PopA_1_, 40 samples from PopA_2_, 20 samples from PopB_1_, 10 samples from PopB_2_ and 5 samples from PopC. The results of NetView were compared with PCA results on the same sample sizes. For the data set containing 40 samples per population, all the individuals of the five populations have still been assigned to their correct sub-populations by Spc (Figure S4A), although the detected clusters were less significant, as the clusters were detected at a lower level of cluster stability and that the clusters were projected in a slightly different neighbourhood within the network visualization (NetView). For the sample size 20 and 10 individuals per population, the clusters became less stable and closely related sub-populations PopB_1_ and PopB_2_ (Spc tree Figure S4B) and PopA_1_ and PopA_2_ (Spc tree Figure S4C) were assigned to single clusters. Visualizing the network structure, which shows population structure in finer detail, it can be seen that sub-structure exists within the closely related populations based on the number of internal links between individuals within the population (NetView in Figure S4B and S4C). However, a clear cluster solution cannot be seen at this depth of sampling. In this context, we also noticed that a value of *k*-NN = 10 is possibly overestimating the genetic distance threshold using data sets with less samples per population, e.g. applying *k*-NN = 3 on the data set with 10 samples per population, all individuals were accurately clustered into 5 populations (Figure S4D).

Horn’s parallel analysis on varying sample size resulted in 4 significant PCs for the data sets containing 40 and 20 samples per population, whilst for the data set with 10 samples per population only 3 significant PCs were retained. Performing *k*-means clustering on the low-dimensional data (4 PCs) of the sub-sets containing 40 and 20 samples per population, all individuals were successfully allocated to their populations, whilst using 10 samples per population only individuals from PopC were correctly clustered to their population. It seems that for a very small sample size PCA performs better than NetView especially when *k*-NN = 10 is used.

Applying NetView to the heterogeneous sample collection, the closely related populations PopA_1_ and PopA_2_ clearly differentiate, whilst PopC and PopB were assigned to one cluster, before they were separated into two distinct populations (Figure S5A). PCA matches the results from NetView, by separating PopA_1_ and PopA_2_ contrasting PCA1 vs PCA2, whilst PCA1 vs PCA3 provide better differentiation between PopC, PopB_1_ and PopB_2_ (Figure S5B). Therefore both PCA and NetView are sensitive to heterogeneous and varying sample size.

#### B: Human HapMap Data set

The human data set used here has previously been described in detail by Pemberton *et al.* [53]. Briefly, it comprises 1,397 samples from 11 populations: 87 African Americans from the South-western United States (ASW); 165 Utah residents with ancestry from Northern and Western Europe (CEU); 137 Han Chinese in Bejing, China (CHB); 109 Chinese in Metropolitan Denver (CHD); 101 Gujarati Indians in Housten, Texas (GIH); 113 Japanese in Tokyo, Japan (JPT); 110 Luhya in Webuye, Kenya (LWK); 86 Mexican Americans in Los Angeles, California (MXL); 184 Maasai in Kinyawa, Kenya (MKK); 102 Toscani in Italy (TSI) and 203 Yoruba in Ibadan, Nigeria (YRI). Eight of these populations (ASW, CEU, CHD, GIH, LWK, MKK, MXL, and YRI) include well documented parent/parent/offspring trios (172) and parent/offspring duos (41) as well as highly related individuals (36 full siblings and 23 half siblings) [53], [64]. For the final computation of ASD between the 1,397 individuals, 1,409,608 autosomal SNP genotypes were used, following the SNP quality controls as described in Pemberton *et al.* [53].

**Figure 4:**
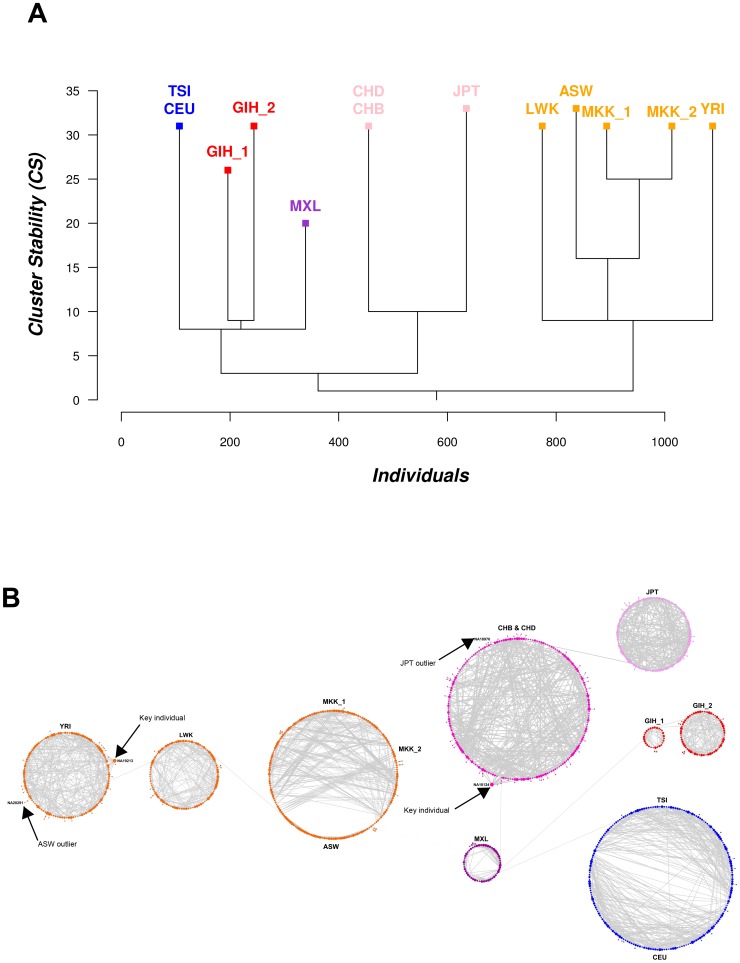
Spc and NetView analysis of human HapMap reference population after removal of closely related individuals. (**A**) Spc tree of clusters representing the grouping of 1,159 unrelated individuals. All individuals have been separated into 11 clusters, representing 9 distinct populations and the existence of sub-structures within GIH and MKK samples. (**B**) NetView of the 1,159 assumed unrelated individuals. The topology of the network highlights the sub-structures within GIH, MXL and MKK and reveals a close relationship between CEU and TSI as well as between ASW and MKK. The identified outliers and key individuals of the population are indicated by their HapMap ID.

**Figure 5:**
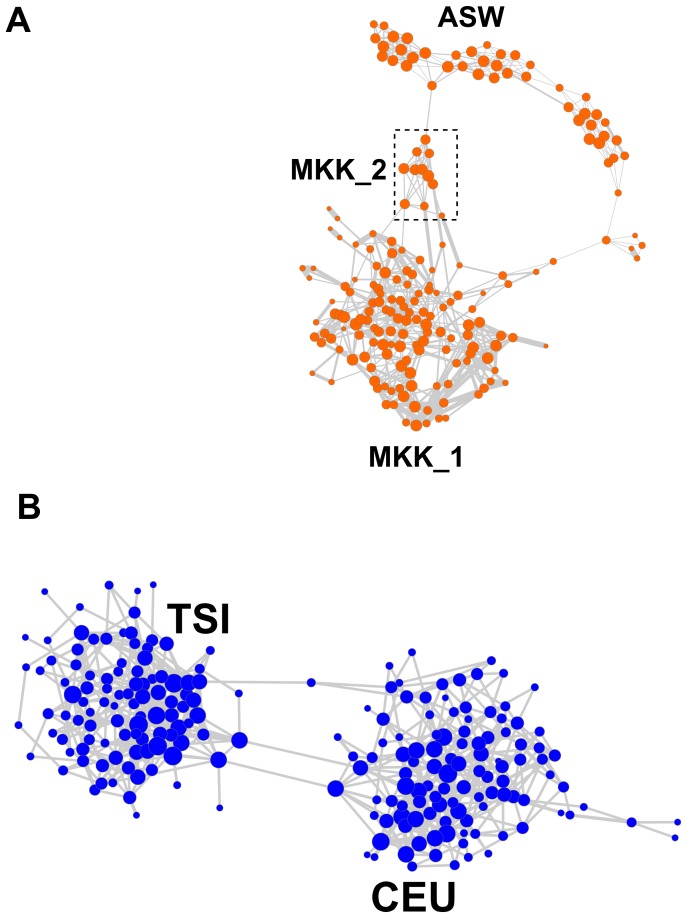
Alternative (organic) NetView of populations with evidence of internal sub-structures. Organic visualization style of (**A**) ASW/MKK and (**B**) TSI/CEU as implemented in software Cytoscape [59]. The network structure of this visualization highlights the existence of sub-structures and clearly identifies cross-linking individuals.

The Spc analysis on the entire human data sets indentified 128 clusters in total, by allocating closely-related individuals (full-siblings, half-siblings, duos and trios) into 116 single clusters (for reasons of over-crowding, the Spc tree is not shown). The network visualization of the Spc analysis on the entire data set highlights the impact of including a large proportion of the previously reported closely related individuals (Figure S6). A few notable features of including such closely-related individuals are that firstly the trio/duo sub-groups form separate mini-clusters among themselves and then are connected to the main cluster of unrelated individuals – see, for example, the “spoke and hub like” characteristics of the CEU and YRI populations in Figure S6. This is also evident in the other populations for closely-related family members forming small individual clusters linked to the main sub-population. Secondly, the internal close relationship amongst related individuals within the MXL, ASW/MKK populations shows evidence of sub-clustering, as shown by the uneven distribution and strength (thickness of edges) of cross links (edges) amongst members of the same population. By contrast, the even distribution of cross links amongst members of the same population (LWK, TSI, CHB/CHD, and JPT) suggests that these members are unrelated individuals. Furthermore, there is also evidence of single members falling outside the main cluster circle of a population, suggesting that these members are less related to its assigned population as a whole, possibly due to admixture or unknown origin. Removal of close genetic links in the test population by excluding progeny from any trio/duo combination resulted in a Spc tree of 11 clusters and is shown in Figure 4A. The hierarchical structure of this tree demonstrates that individuals from populations with South Asian, American, European, East Asian and African ancestry form distinct clusters and are in agreement with previous analyses on the HGDP-CEPH panel and release 2 of phase III of the HapMap using MDS [53] and model-based algorithm *frappe* [65]. In addition, it is now evident that two sub-structures exist within GIH and MKK population. The NetView of this analysis is shown in Figure 4B where 9 distinct clusters are evident. A key feature in the topology of the network can readily be noted that the GIH population consists of two major-well defined sub-populations and that the internal relationship amongst members of the MXL population remains evident after removal of the trio relationships, suggesting unknown internal relationships. The NetView analysis also shows that both the TSI/CEU population and the MKK/ASW are visualized into two single networks, respectively, with evidence of internal sub-structures in these populations. Under an alternative (organic) network visualization option, NetView highlights the population sub-structures within ASW and MKK population (Figure 5A) and clearly identifies individuals cross-linking the TSI and CEU population (Figure 5B).

The relatedness of individuals within the population is clearly visible by NetView, for instance the strong relatedness of individual NA18124 in the Han Chinese population and individual NA19213 in the YRI population is shown in Figure 4B, with 16 and 15 population-wide links, respectively. We consider these individuals as significant since there were no close (kinship) relationships evident for these individuals. This feature is not readily seen in conventional population analyses such as model-based approaches (e.g. Structure), where information on individuals is not captured to this level of detail [8]. This level of detail on individual members shows an outlier previously reported by Pemberton *et al.* [53] within the JPT population (NA18976) found to be connected to two Chinese individuals sampled in Denver (CHD) and a previously unreported outlier African American individual (NA20291) which was found to be connected to the YRI population through a single link (Figure 4B).

#### C: Bovine HapMap Data set

Finally, we used a data set on multiple breeds of cattle which consisted of a total of 501 animals, representing 12 *Bos taurus* breeds (N = 331) with European origin, 2 *Bos taurus breeds* (N = 45) with African origin, three *Bos indicus* breeds (N = 73) and 2 *admixed breeds* (N = 48), as well as two buffalo breeds namely *Bubalos quarelis* (N = 2) and *Bubalus bubalis* (N = 2). A detailed description of the breeds and samples used by the Bovine HapMap consortium is given in [31] and is also listed in Table S1. The animals within each breed were documented to be unrelated for ≥4 generations. It has been documented that each breed includes one or two sire/dam duos and at least one sire/dam/offspring trio. For 8 of the 14 breeds, samples were sourced from multiple geographical locations as detailed in Table S1. All samples were genotyped using the 50K Illumina Bovine BeadChip. For the present analysis we included samples with a genotype call rate >0.9 and excluded the 4 buffalo samples. The final bovine data set used in this study includes 477 animals from 19 genetically distinct sub-populations with genotypes on 32,653 autosomal SNPs.

**Figure 6:**
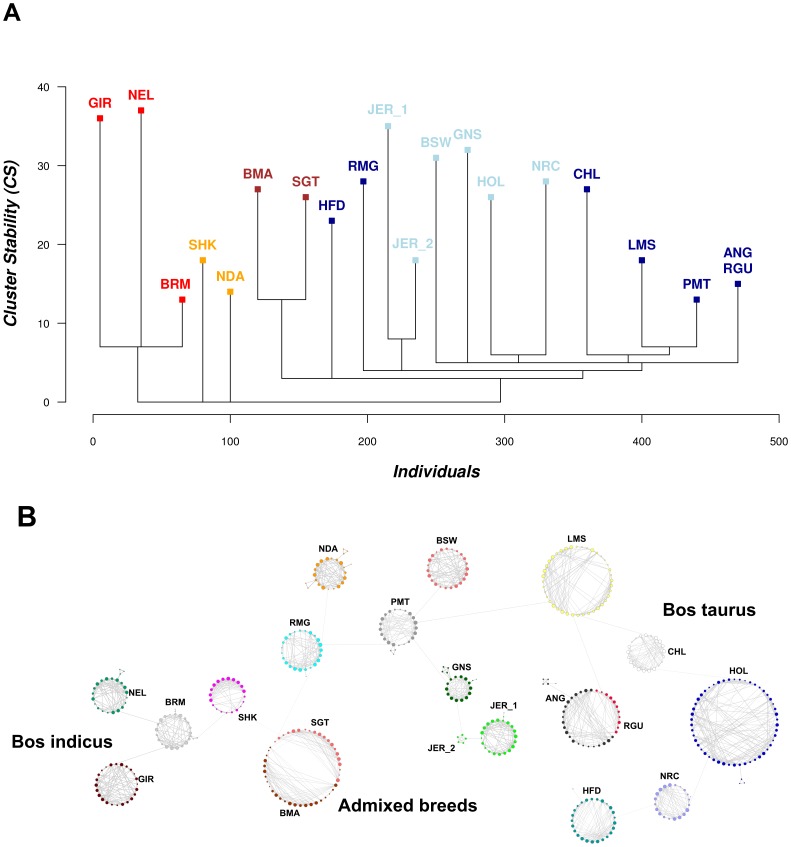
Spc and NetView analysis of the Bovine HapMap data. (**A**) Spc tree of clusters representing the grouping of 477 animals represented in the Bovine HapMap data set [31]. The animals have been allocated into 19 clusters, representing 18 out of 19 breeds and the existence of sub-structures within JER (JER_1 and JER_2), and a merged Angus cluster (ANG and RGU). (**B**) NetView of 477 bovine HapMap samples from *Bos taurus*, *Bos indicus* and *admixed origins*. The topology of the network reflects the genetic relatedness between cattle breeds and reveals sub-structures within LMS, SHK and ANG cluster.

The Spc analysis of the entire bovine data set successfully allocated all animals to their respective breed. For the JER breed group, animals were separated in two clusters (JER_1 and JER_2) based on the geographical origin of the samples, whilst for the RGU breed group; all animals were clustered with the ANG cluster (breed group). The hierarchical structure of the Spc tree reflects that cattle breeds are highly structured according to sub-species (*Bos taurus* vs *Bos indicus*), geographic origin (African vs Indian, vs European) and primary purpose (dairy or beef) (Figure 6A). At the base of the tree, the most readily differentiated sub-clustering occurred between *Bos indicus* and European *Bos taurus*, with further evidence of African taurine (NDA) and an ancestral indicine/taurine hybrid (SHK) forming separate breeds groups. Within the European taurine based groups, the two *admixed breeds* (SGT and BMA, both with European taurine content) clearly separate out from the remaining European taurine breeds. This tree is in broad agreement with the Structure and PCA results reported by the Bovine HapMap group who have generated the data [31].

Also evident are the different levels of cluster stability (CS) at which each cluster separates, which suggests that different sub-structures exist within the European cattle breeds and that these breeds are less related to each other, compared to *Bos indicus* and *admixed breeds* possibly as a result of SNP-discovery ascertainment bias [11]. The final position of the clusters suggests that the GIR and NEL samples are highly related, whilst BRM, LMS, PTM and ANG cluster out at lower positions, indicating the existence of sub-structures or co-relationships with other breeds. The network visualization of the bovine data highlights the transitional position of the *admixed breeds* SGT/BMA and SHK between the *Bos indicus* breeds and the *Bos taurus* breeds (Figure 6B). Furthermore, the network visualization also shows the transitional position of breeds within the *Bos indicus* and *Bos taurus* breeds groups (i.e. BRM, PTM and LMS), as shown with the highest numbers of external links to other breeds. The network structure within breeds additionally reveals sub-structures within the LMS, ANG, and SHK clusters. Within LMS the sub-structures may arise due to different origins of the samples, where samples have been taken from USA and France. Within the ANG, the network identifies RGU samples as a sub-population of the ANG breed, whilst the sub-structure within SHK may indicate samples of different origin or heterogeneous sample collection. Other breeds which include samples of different origin show that in extreme cases such as JER the samples from different origins cluster as two separate clusters but with a close connection between them. The network visualization also highlights heterogeneity in sample relationships within breeds, as evidenced by mini-clusters of closely related samples and small sub-groups within the main breed cluster, i.e. GNS, BRM, NEL, PMT and NDA. These high-resolution features of sub-structure are not readily evident from PCA results and Structure analyses shown by the Bovine HapMap consortium [31] and highlight unique attractive features offered by NetView.

## Discussion

We have introduced a network-based clustering procedure (Spc) to population genetic analysis to infer high-resolution population structure without any *a priori* ancestry information from high density genotypic data. Furthermore, we combined Spc results with network visualisation tools into an analysis pipeline called “NetView” for high definition network visualization. We compared our results with two popular methods namely non-parametric (e.g. PCA [15]) and parametric approaches (e.g. Admixture [18]). An overall comparison of the three presented approaches including the different input parameters and the various output styles are provided in Table 1.

**Table 1:** Overall comparison of the three different approaches currently applied to study genome-wide population structures.

Name	Applicability	Performance
**NetView**	Genotype or Haplotype based	Determines population clusters based on
	distance matrix	*k*-nearest neighbors (*k*-NN).
		Creates a hierarchical structure of
		population clusters.
		Provides a high-definition network
		visualization, which allows the identification
		of fine-scale population structure and closely
		related individuals (duos and trios).
		Visualizes the relatedness of individuals
		within populations.
		Can also be used to determine genome-wide
		membership proportions (unpublished result).
	
	**Notes:** The cluster performance is strongly dependent on the prior choice of *k*-NN.	
	It can be recommended to start with *k*-NN = 10 and also explore other values.	
	There are also other tuning parameters (Delta T and number of pott spins), but	
	they are not as important as the *k*-NN criterion. There is no appropriate method to	
	determine the optimal number of *k*-NN.	
**Non-parametric**	Any similarity matrix or genotype	Creates a visualization of fine-scale
**(PCA)**	matrix (Individuals/SNPs)	population structure in 2 or 3 dimensional
		space.
		Calculates the variation which is explained
		by each principal component (PC).
		Determines the optimal number of K.
		
	**Notes:** To cluster individuals to respective populations and to determine the	
	optimal number of clusters (K), PCA has to be applied in combination with a	
	clustering tool e.g. *k*-means. To provide an optimal visualization and interpretation	
	of the cluster result, the number of significant PCs has to be determined with a test	
	statistic e.g. Horns parallel analysis (PA). The visualization is limited to 3D-space.	
**Parametric**	Genotype matrix	Computes individual membership
**(Admixture)**		proportions between individuals for any
		given number of clusters (K).
		Identifies admixed individuals.
		Determines the optimal number of K with an
		implemented cross-validation procedure.
		Provides a hierarchical population structure
		and F_ST_ estimates between the populations.
	**Notes:** Needs a data-validation check of the relatedness between individuals.	
	Takes different model parameters as input. Determines the number of clusters with	
	a cross-validation procedure, which is not appropriate for all data sets, e.g.	
	simulated population structure results. Provides no convergence for cluster runs	
	when applied to data sets with K >10. Compared to the other two methods, this	
	procedure gets computational demanding when applied to full sequenced data with	
	millions of polymorphic sites.	

In order to evaluate the efficiency of NetView to detect fine-scale population structure, we have compared our findings across three previously published data sets. For the simulated data set, PCA coupled with *k*-means clustering performed well to separate the samples into distinct genetic groups, using four significant PCs and a predetermined optimal number of K. Admixture could neither determine the correct number of clusters nor correctly separate close related sub-populations that would have suggested recent admixture. By contrast Spc analyses of the same data revealed a hierarchical structure between groups that perfectly corresponded to the known phylogenetic relationship between them. Given the network structure in NetView which is derived through the clustering procedure, additional information on the relatedness of individuals within their respective populations can be shown. For instance important key individuals within groups and the position of less related individuals can be readily visualized. The combination of all these findings implies that populations can be structured into different sub-populations on a very fine scale.

The real human and bovine genotype data presented in this study, describe two different population structures. The human population structure represents a population with distinct groups; where samples have descended from a few well-differentiated ancestral populations as a discrete grouping. The bovine data exemplifies a more continuous population structure with many closely related sub-populations described as breed and admixed breeds. Model based approaches like Admixture are more effective in the characterisation of distinct groupings, whilst PCA based approaches perform better on continuous groupings [66]. Applying Spc on the two different types of data sets clearly demonstrates the efficiency of this approach to handle the combination of discrete and continuous sub-populations. This is effectively shown by not imposing a partition on the European population (CEU and TSI) and Angus breeds (ANG and RGU) when there are no natural sub-populations present. This is consistent with recent findings by Gusev *et al.* [67] who also could not demonstrate separation between CEU and TSI populations based on genome wide IBD sharing. Iterative removal of individuals with strong cross links subsequently resulted in two distinct populations by Gusev *et al.* [67] and confirms our findings under NetView (Figure 5B). In this context, we have demonstrated the ability of NetView to reveal unknown fine-scale population sub-structures (e.g. GIH, MKK and JER) and to identify closely related (admixed) individuals between populations (e.g. CEU and TSI), which is useful for the design of genome-wide association studies [1]. The network visualization of the human data set shows that NetView additionally provides fine-scale population sub-structures, and reveals information about each individual simultaneously, detecting important key individuals and closely related individuals (trios and duos) as well as family structures (e.g. MXL). In this context, we have demonstrated that NetView is a useful tool to detect unexpected genetic relatedness among samples and incorrect sample assignment, knowledge of which is important in the interpretation of phylogenetic studies [68]. As such NetView can be used as an editing tool to eliminate such individuals from population studies, when unrelated samples are required as in the case of Admixture and Structure. Application of NetView can also demonstrate inappropriate sample collection in phylogenetic studies when small and inadequate sample sizes are selected for population analyses as shown in the analysis of the simulated data set.

In this study we observed that *k*-NN = 10, seemed to be a reasonable starting point for most analyses. However, we have also noted that for this number of *k*-NN, experimental designs including less than 20 samples per population will be of limited value. Hence, for such small data sets per population, we suggest to start with *k*-NN = 10 and additionally re-run NetView with a smaller number of *k*-NN, simultaneously investigating different cluster solutions. Step-wise reduction of the number of *k*-NN allows examination of the population structure at different levels of resolution and simultaneously describes a zoom in effect as shown on the simulated data set, whilst increasing *k*-NN will allow for a more detailed analysis of admixture between populations.

We believe that network analyses can especially be useful to study the population structures of indigenous breeds and wild species where population information is often lacking, hereby providing essential information for conservation and genetic resource management decisions applicable to any species [69], [70]. NetView provides the means to detect putative important founder/key individuals within populations and to accurately study gene flow between populations [41], [42]. These properties of network visualization and analysis can be useful in the characterization of managed and non-managed populations. The results in this study show that it is possible to detect fine-scale population structures only from genetic relationships.

NetView can be applied to livestock (cattle, pigs and sheep), domestic animals (dog, cat and horse) and their related wildlife ancestral populations where it is now feasible to collect and analyse extensive global sample banks for genome wide SNP content on vertebrate species (http://www.genome10k.org/) [71] and other organisms.

As we discussed earlier different genetic relationship or distance matrices can be used in the NetView analysis. For a meaningful comparison of the results presented in this study we use previously applied genotype matrix (IBS) for the two real data sets and shared haplotypes in case of the simulated data set. However, the effect of the type of relationship matrix, SNP density, minor allelic frequencies and use of haplotype information on the performance of NetView require further investigations. With the availability of next generation sequencing data from large number of sequenced and non-sequenced species, it would be possible to compute sequence based genetic relationship matrix and hence allows the application of NetView to such data sets.

Future applications of NetView can also be extended to high resolution analysis of admixture which is common in many human and animal populations [31], [65]. The analysis can be done to detect the systematic and planned occurrence of admixture as demonstrated in the formation of multi-breed cross bred populations in livestock to capture the benefit of heterosis, or the spontaneous occurrence of admixture in population interactions as seen in human populations. NetView can reveal admixture on a population basis or down to individuals, and may be extended to chromosome or sub-chromosome level to examine selective and adaptive fitness. NetView may also be extended to incorporate long and short range haplotype information, thereby capturing historical events on the formation of populations as has been done in humans [72] and sheep [73].

## Supporting Information

Figure S1 **Workflow of NetView.** Schematically representation of the 5 different components included in the Netview procedure.
(PDF)

Figure S2 **Calinski Criterion values plotted for each incremental value of K, increasing K form 2 to 7.** The modal value of this distribution corresponds to the true K(*) or the uppermost level of structure for the simulated data set, here K = 5.
(PDF)

Figure S3 **Cross-validation error plotted obtained through Admixture for each incremental value of K for the simulated data set.**
(PDF)

Figure S4 **NetView application on reduced numbers of individuals.** Spc trees of clusters representing the groupings of individuals with common applied *k*-NN = 10 and corresponding network visualizations considering **(A)** 40, **(B)** 20 and **(C)** 10 samples per population. **(D)** Spc tree and network visualization of 10 samples per population with *k*-NN = 3.
(PDF)

Figure S5 **Analysis of heterogeneous sample collection.** **(A)** Spc tree and network visualization of the heterogeneous sample collection including 80, 40, 20, 10 and 5 individuals form population PopA_1_, PopB_1_, PopA_2_, PopB_2_ and PopC respectively. **(B)** PCA scatter plots of the heterogeneous sample collection, contrasting PC1 vs PC2 and PC1 vs PC3.
(PDF)

Figure S6 **High-definition network visualization (NetView) of 1,397 individuals from 11 reference populations represented in the human HapMap data set** [**53**] **.** Each individual is represented by a node; with the different shades denote the sample origin. The thickness of edges, which varies in the proportion to the genetic distance, has been used to visualize individual relationships within and between populations. The node size, which varies in proportion to the numbers of edges per node, illustrates how well each individual is connected within the population. The determined mini-clusters of close and less related individuals are indicated by solid circles. In addition to the main cluster network, duo and trio relationships are shown as well as unrelated individuals.
(PDF)

Text S1 **Detailed description of network construction and clustering procedure.**
(DOC)

Table S1 **Summary of sampled cattle populations represented in the bovine HapMap data set** [**31**] **.** Breed origin, country of sampling and breed characteristics are indicated, together with the number of sampled animals included in this study (N).
(DOC)
