# Supplementary figures and images for: NetView: A High-Definition Network-Visualization Approach to Detect Fine-Scale Population Structures from Genome-Wide Patterns of Variation
Source: PLoS One. 2012 Oct 31;7(10):e48375. doi: 10.1371/journal.pone.0048375 (PMC3485224; doi:10.1371/journal.pone.0048375)

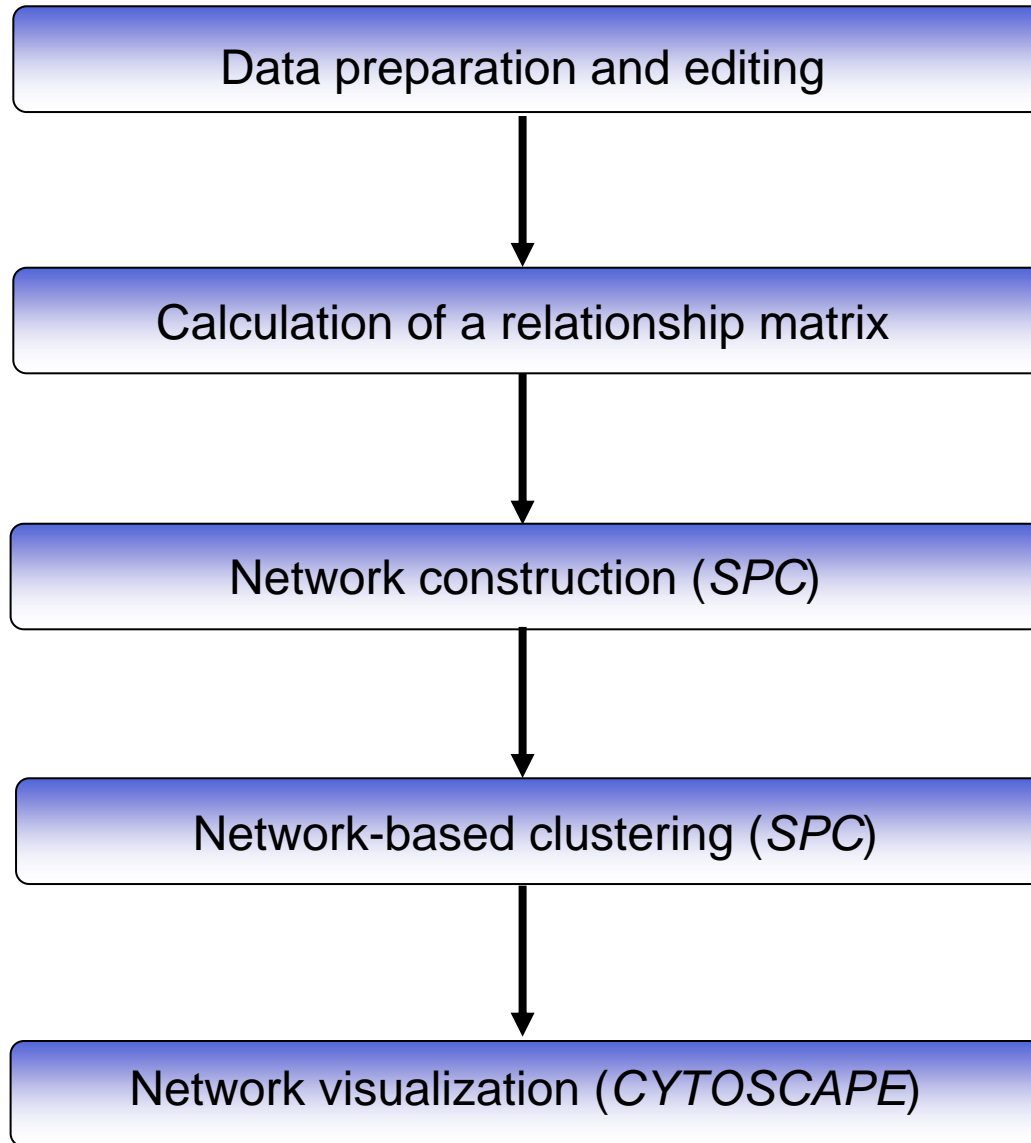

Supplement: Figure S1 — Workflow of NetView. Schematically representation of the 5 different components included in the Netview procedure. (PDF) [file pone.0048375.s001.pdf]

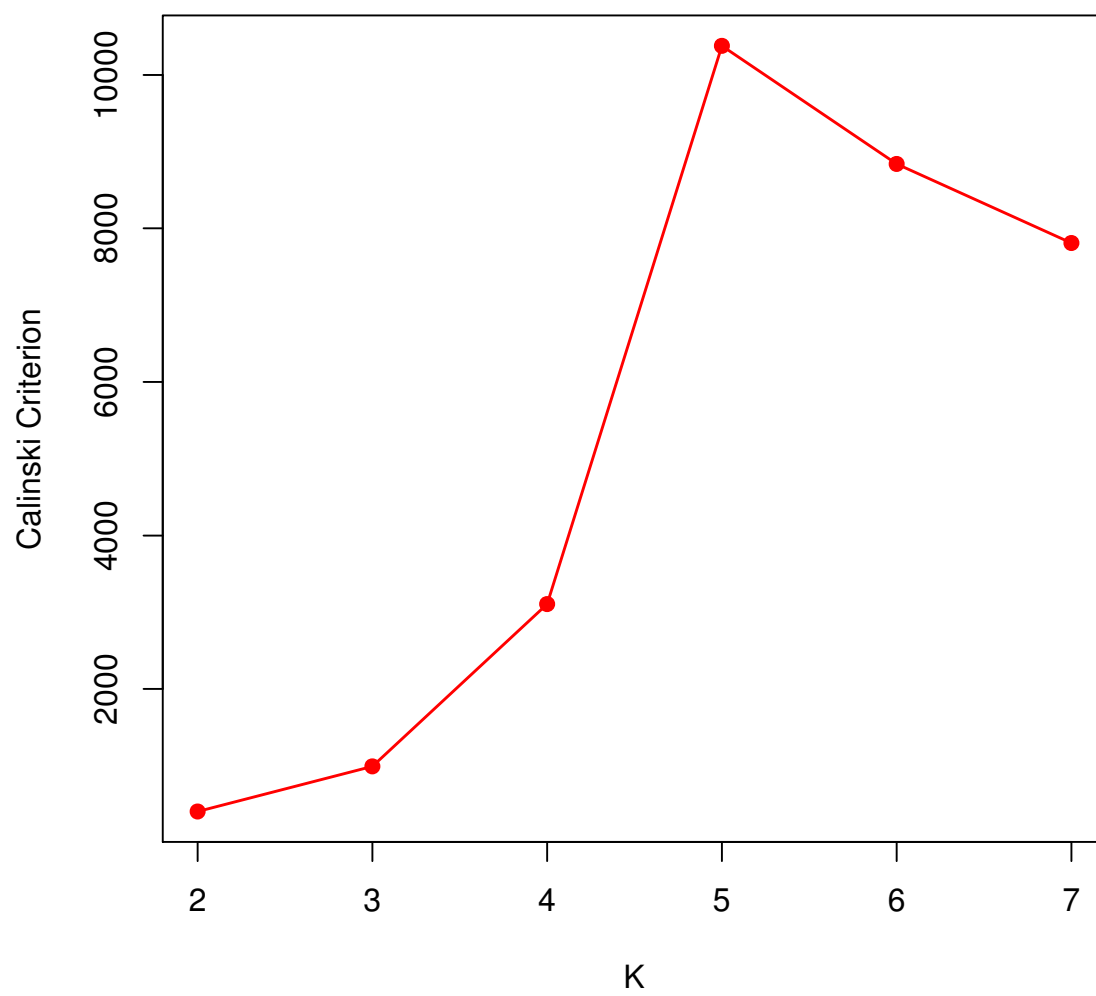

Supplement: Figure S2 — Calinski Criterion values plotted for each incremental value of K, increasing K form 2 to 7. The modal value of this distribution corresponds to the true K(*) or the uppermost level of structure for the simulated data set, here K = 5. (PDF) [file pone.0048375.s002.pdf]

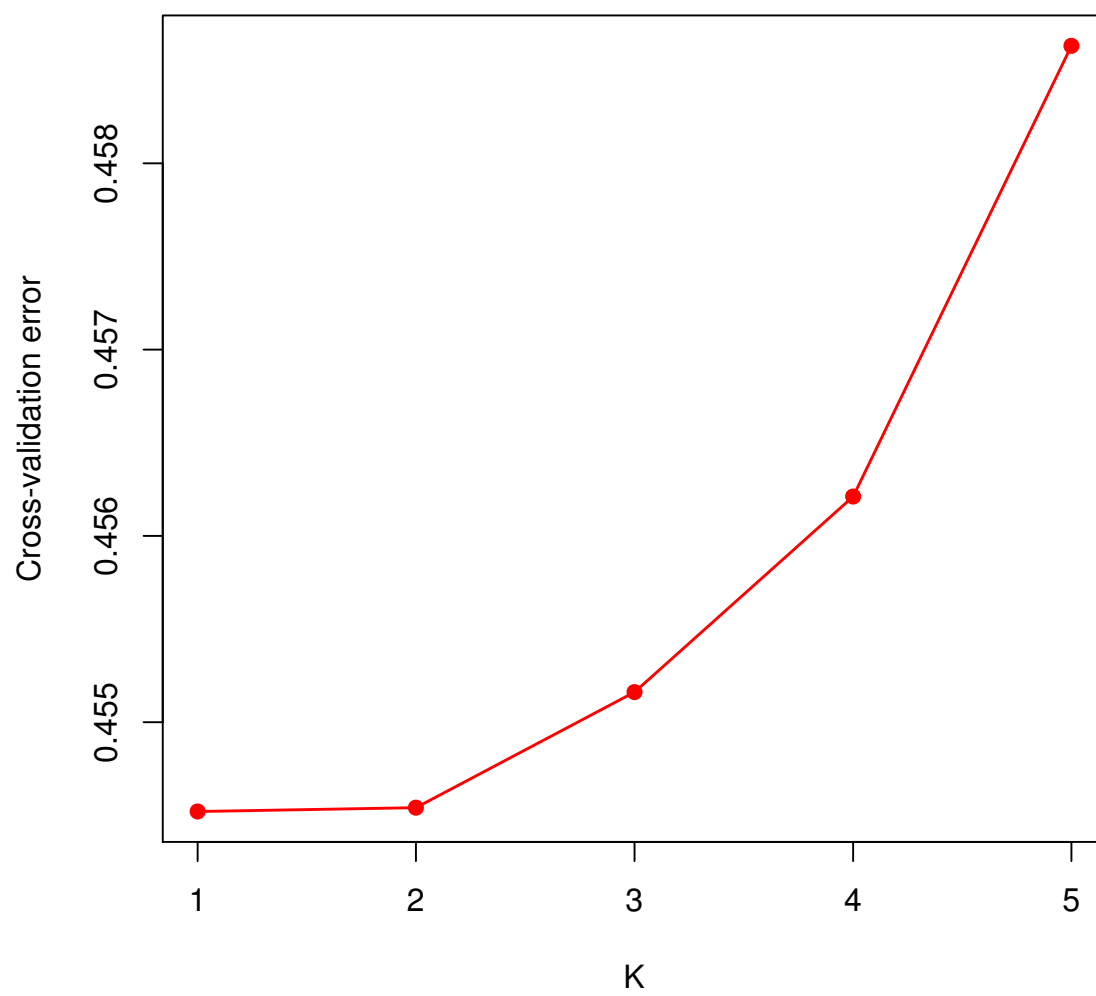

Supplement: Figure S3 — Cross-validation error plotted obtained through Admixture for each incremental value of K for the simulated data set. (PDF) [file pone.0048375.s003.pdf]

**A**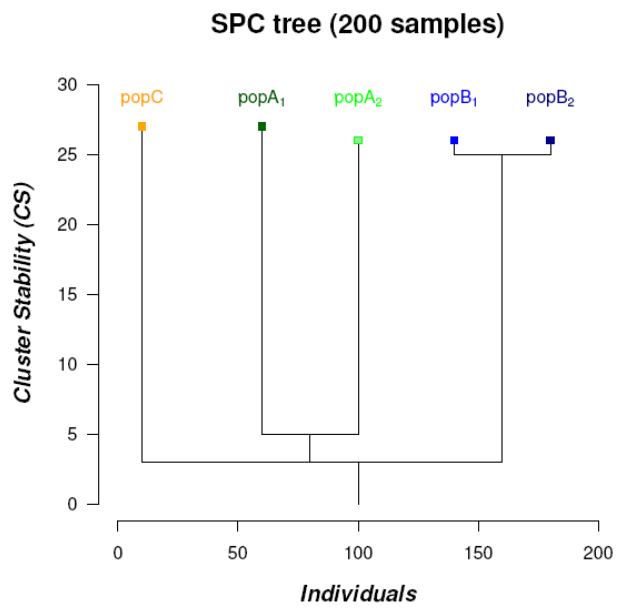**NETVIEW**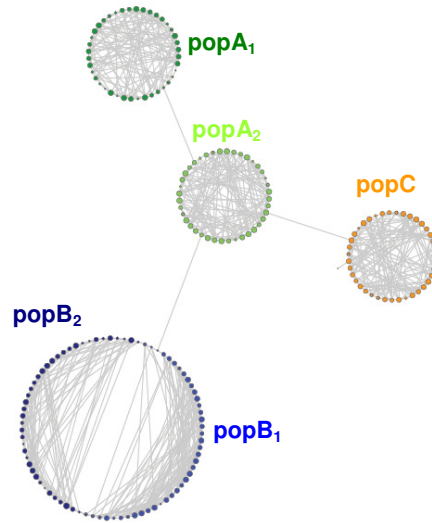**B**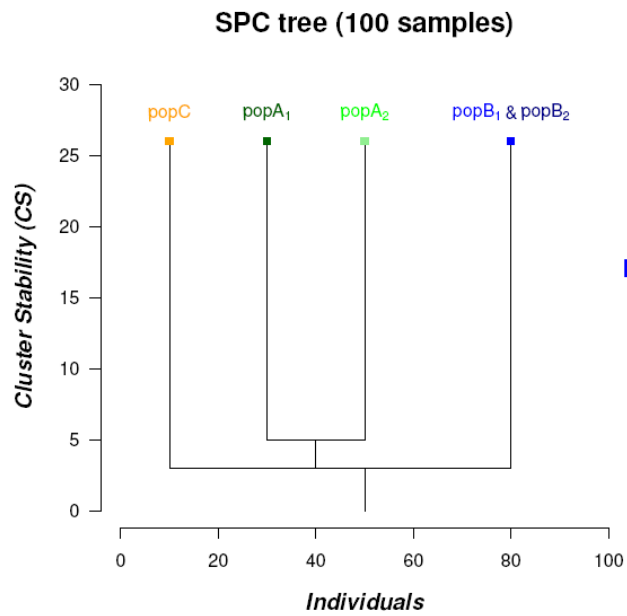**NETVIEW**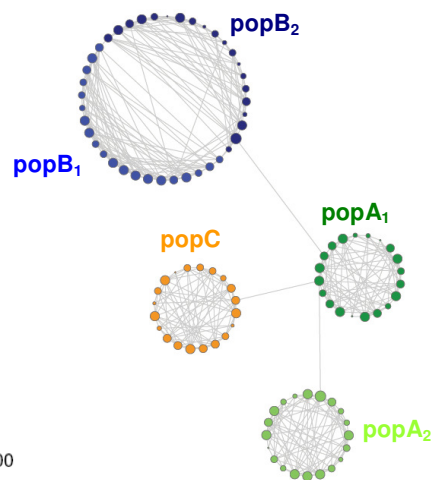

**C**

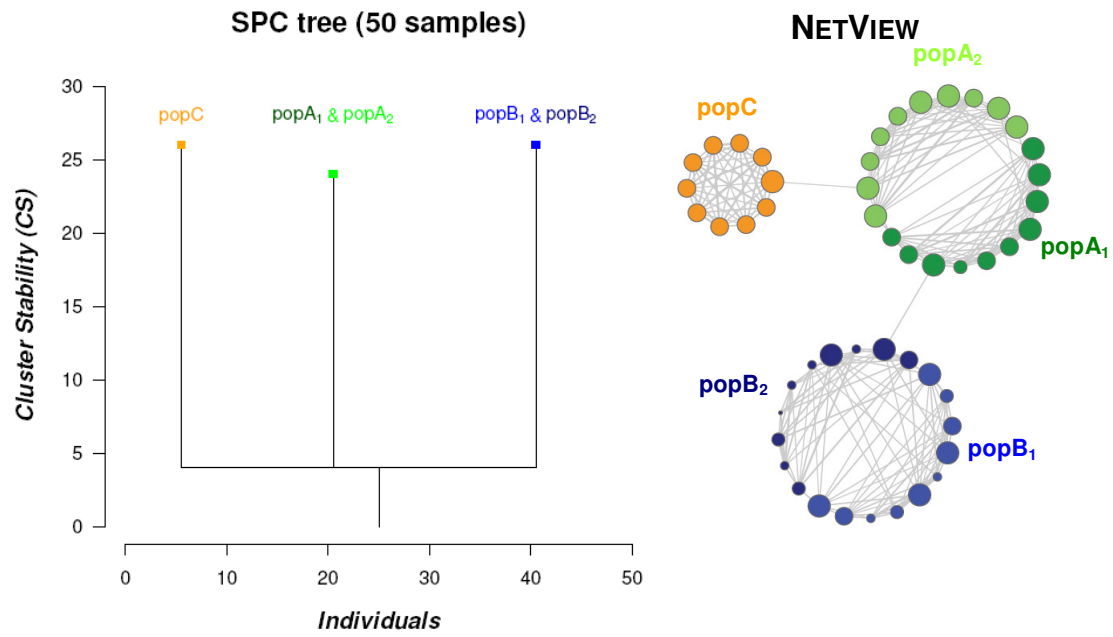

**D**

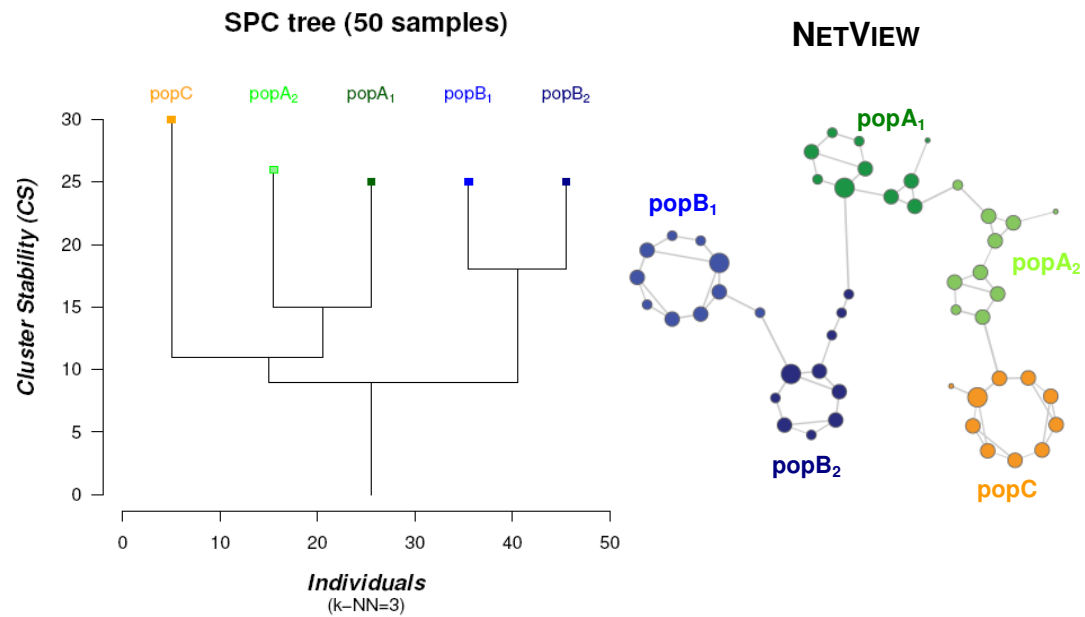

Supplement: Figure S4 — NetView application on reduced numbers of individuals. Spc trees of clusters representing the groupings of individuals with common applied k-NN = 10 and corresponding network visualizations considering (A) 40, (B) 20 and (C) 10 samples per population. (D) Spc tree and network visualization of 10 samples per population with k-NN = 3. (PDF) [file pone.0048375.s004.pdf]

**A**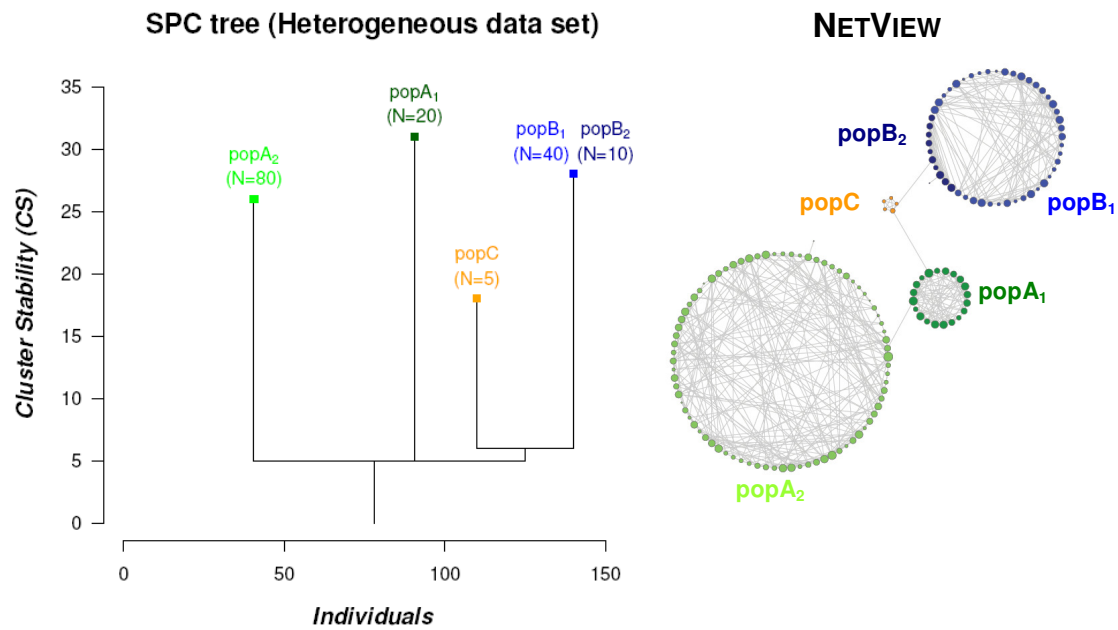**B**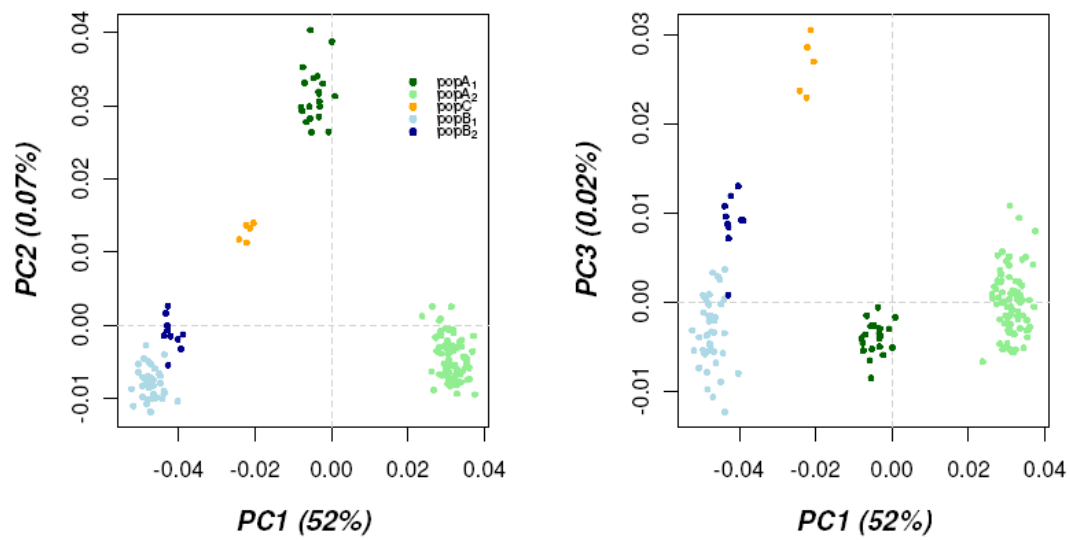

Supplement: Figure S5 — Analysis of heterogeneous sample collection. (A) Spc tree and network visualization of the heterogeneous sample collection including 80, 40, 20, 10 and 5 individuals form population PopA1, PopB1, PopA2, PopB2 and PopC respectively. (B) PCA scatter plots of the heterogeneous sample collection, contrasting PC1 vs PC2 and PC1 vs PC3. (PDF) [file pone.0048375.s005.pdf]

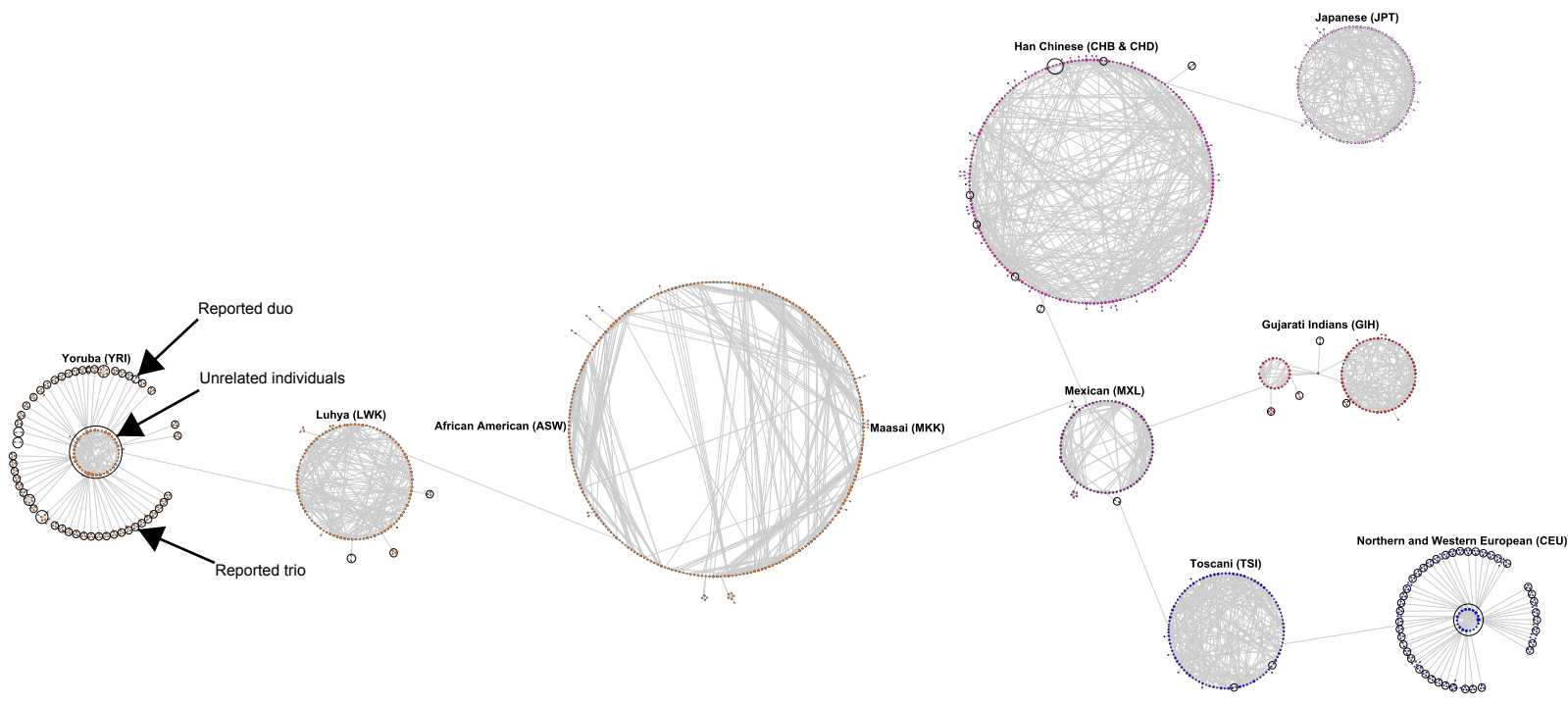

Supplement: Figure S6 — High-definition network visualization (NetView) of 1,397 individuals from 11 reference populations represented in the human HapMap data set [53] . Each individual is represented by a node; with the different shades denote the sample origin. The thickness of edges, which varies in the proportion to the genetic distance, has been used to visualize individual relationships within and between populations. The node size, which varies in proportion to the numbers of edges per node, illustrates how well each individual is connected within the population. The determined mini-clusters of close and less related individuals are indicated by solid circles. In addition to the main cluster network, duo and trio relationships are shown as well as unrelated individuals. (PDF) [file pone.0048375.s006.pdf]
