# Supplementary material for: NetView: A High-Definition Network-Visualization Approach to Detect Fine-Scale Population Structures from Genome-Wide Patterns of Variation
Source: PLoS One. 2012 Oct 31;7(10):e48375. doi: 10.1371/journal.pone.0048375 (PMC3485224; doi:10.1371/journal.pone.0048375)
Supplement: Text S1 — Detailed description of network construction and clustering procedure. (DOC) [file pone.0048375.s007.doc]

**Text S1** Detailed description of network construction and clustering procedure.

*1. Network construction*

To create a network from a pair-wise distance matrix (1 – relationship) between individuals, a three-step procedure is applied. **(i)** For each individual *X_i_*, *i* = 1,…,*N*, make a list (*L_i_)* of its first *k* nearest neighbours (*k*-NN), specifying the maximum number of nearest neighbours an individual can have. **(ii)** Connect all individuals *X_i_* and *X_j_* through an edge if $i \in L_{j}$ and $j \in L_{i}$, otherwise the individuals are not connected. **(iii)** Individuals not connected to any other individual following the *k*-NN criterion, are then connected to its nearest neighbours according to a minimum spanning tree associated with the data .

*2. Cluster procedure*

After the network construction, the clustering is performed based on the physical framework of a Potts model simulation. The Potts model simulation describes the physical behaviour (state) of inhomogeneous ferromagnetic particles along a simulated temperature (T) gradient (this is analogous to the genetic relationship in this study). To describe the physical behaviour between particles along varying conditions (e.g. thresholds of genetic distances) each data point in the pre-defined network gets associated with a so-called Potts spin variable *s*. Values of *s* randomly take one of *q* integer values (*s* = 1,2,….*q*) to distinguish between different conditions at which Spc is applied. Since the clustering result is insensitive to the value of *q* , we have worked with a commonly applied *q* value of 20 . Subsequently, each Potts spin variable associated with data points X*_i_* and X*_j_* are coupled by an interaction of strength indicated by a coupling constant *J_ij_* > 0. The coupling constant *J_ij_* is a positive decreasing function of genetic distance (*D_ij_*) between X*_i_* and X*_j_*, the population-wide average number of (*K*) interacting neighbours, and their mean distance ($\bar{D}$). J*_ij_* is expressed as:

(1.1)

Choosing *J_ij_* this way creates strong interactions between spins (*s*) associated with individuals having high relationships, and weak interactions between individuals having fewer relationships. The cluster formation of data points along simulated sequences of T, is an assignment of values $S = \left\{s_{i}\right\}_{i=1}^{N}$. For each assignment, the Hamiltonian cost function H(S) is defined as follows:

$$H(S) = -\sum_{ij} J_{ij} G_{sisj} .$$

(1.2)

Where, as shown in the equation above (1.2), the assignment of values *S* is determined by the so-called spin-spin correlation function (G*_sisj_*) and coupling constant J*_ij_* (1.1). For the final cluster assignment of individuals the correlation between neighbour spins G_sisj_ for all pair-wise combination of individuals as defined in the population network is calculated. To calculate this correlation function for a range of 0 ≤ T < T_max_ the magnetization of each spin as a function of the temperature is calculated using mean field approximation [58], which in general describes a density function,

$$G_{sisj }= \frac{(q-1)m^{2} + 1}{q} .$$

(1.3)

To determine the global minimum of the Hamiltonian cost function (1.2), the magnetization *(m^2^)* of spins is determined in an iterative search along T. With increasing T the system passes through three different phases namely ferromagnetic, paramagnetic and super-paramagnetic. Ferromagnetic and paramagnetic states describe the extremities of the system e.g. at very low T, individual spins have strong interactions with their neighbours and all data stay in one cluster the system is therefore in the ferromagnetic state, whilst the paramagnetic state describes the opposite, high temperature and low spin-spin correlation function among neighbours, causing many clusters. The super-paramagnetic state is the intermediate, where the magnetizations at critical T_cij_ of spins (1.3) reach a global minimum of the Hamiltonian cost function (1.2) and the true numbers of clusters appear. The final hierarchical structure of clusters summarizes the clustering result at each temperature step, where the cluster core of the true number of clusters can be expanded to build larger and less stable clusters. In this context, the range of temperatures ∆T = T_2_ – T_1_ a cluster splits form its parent is used to quantify the stability of clusters (*CS*) and the relationship between them.

Applying Spc to population genetics is substantially different to classical algorithms for construction of phylogenetic networks/trees based on individual distances e.g. Neighbor Joining (NJ) algorithm introduced by Saitou and Nei [68]. NJ algorithm begins with a star tree, which is produced under the assumption that there is no clustering of data points and aims to find true neighbours (two data points that are connected by a single edge in an unrooted tree) by minimizing the sum of all branch lengths. Therefore, NJ algorithm starts with the closest and finishes with most distant neighbours. Contrary to NJ algorithm, Spc starts at T = 0, where all data points form one cluster. This initial hypothetical taxonomic unit splits successively within a continuous threshold of genetic distance. Thereby, the splitting occurs in an opposite order compared to the NJ algorithm, i.e. Spc algorithm starts to split most distant composed data objects hereby producing a hierarchical structure of clusters.
