# Supplementary material for: NetView: A High-Definition Network-Visualization Approach to Detect Fine-Scale Population Structures from Genome-Wide Patterns of Variation
Source: PLoS One. 2012 Oct 31;7(10):e48375. doi: 10.1371/journal.pone.0048375 (PMC3485224; doi:10.1371/journal.pone.0048375)
Supplement: Table S1 — Summary of sampled cattle populations represented in the bovine HapMap data set [31] . Breed origin, country of sampling and breed characteristics are indicated, together with the number of sampled animals included in this study (N). (DOC) [file pone.0048375.s008.doc]

**Table S1** Summary of sampled cattle populations represented in the bovine HapMap data set [31]. Breed origin, country of sampling and breed characteristics are indicated, together with the number of sampled animals included in this study (N).

| **Breeds** | **Breed code** | **N** | **Country of sampling** | **Characteristics** | **Origin** |
| --- | --- | --- | --- | --- | --- |
| *Bos taurus* |  |  |  |  |  |
| Angus | ANG | 27 | USA and NZ | Black coat  Meat quality | Scotland |
| Brown Swiss | BSW | 23 | USA | Brown coat  Rugged appearance | Switzerland |
| Charolais | CHL | 21 | USA | White to cream coat  Large body size | France |
| Guernsey | GNS | 20 | USA and UK | Tan and white coat  Refined structure | Channel Islands |
| Hereford | HFD | 26 | USA and NZ | Read coat with white face | UK |
| Holstein | HOL | 53 | USA and NZ | Black and white coat  High milk yield | Netherlands |
| Jersey | JER | 27 | USA and NZ | Small size  Milk quality | Channel Islands |
| Limousin | LMS | 40 | USA and France | Red coat  Muscularity | France |
| N’Dama | NDA | 24 | Guinea | Fawn coat, small size  Trypanosome resistance | West Africa |
| Norwegian Red | NRC | 21 | Norway | Red and white coat  High milk yield | Norway |
| Piedmontese | PMT | 24 | Italy | Gray coat, dark skin Muscularity | Italy |
| Red Angus | RGU | 11 | USA and Canada | Red coat, Meat quality | Scotland |
| Romagnola | RMG | 24 | Italy | Ivory to gray coat, black skin, Muscularity | Italy |
| Sheko | SHK | 20 | Ethiopia | Small size, Brown coat of variable shades  Trypanosome resistance | Ethiopia  (East Africa) |
| *Bos indicus* |  |  |  |  |  |
| Brahman | BRM | 25 | USA and Australia | Gray coat, humped,  heat tolerance | USA |
| Gir | GIR | 24 | Brazil | Mottled red and white coat, humped, heat tolerant | India |
| Nelore | NEL | 23 | Brazil | White to gray coat, humped, heat resistance | India |
| *Admixed Breeds* |  |  |  |  |  |
| Beefmaster | BMA | 24 | USA | Variable, predominantly red coat, robustness | USA |
| Santa Gertrudis | SGT | 20 | USA | Red coat, drought, heat, insect an disease tolerance | USA |
